# Adverse childhood experiences and use of corporal punishment among women in low-resource settings: a convergent mixed methods study with mothers of children under five in the Dominican Republic

**DOI:** 10.1186/s12905-025-03742-y

**Published:** 2025-12-03

**Authors:** Adrianne Katrina Nelson, Melanie P. Frías, Martha Vibbert, Carl Kendall, Laura Sánchez-Vincitore, Heidi Luft, Michelle M. Susana, Katherine Theall, Arachu Castro

**Affiliations:** 1 Yale School of Public Health, Department of Health Policy and Management, 60 College St, New Haven, CT 06510 USA; 2 Universidad Iberoamericana, Laboratorio de Neurocognición y Psicofisiología (NeuroLab), Calle Majoma No. 13, Los Ríos, Santo Domingo, República Dominicana; 3 Boston University Chobanian and Avedisian School of Medicine, 72 East Concord St, Boston, MA 02118 USA; 4 Celia Scott Weatherhead School of Public Health and Tropical Medicine at Tulane University, Department of Social, Behavioral, and Population Sciences, 1440 Canal St, New Orleans, LA 70112 USA; 5 Federal University of Ceará, College of Medicine, Department of Community Health, Avenida da Universidade, Benfica, Fortaleza, CE 2853 Brazil; 6 University of Texas Medical Branch, School of Nursing, 301 University Boulevard, Galveston, TX 77555 USA; 7 Celia Scott Weatherhead School of Public Health and Tropical Medicine at Tulane University, Department of International Health and Sustainable Development, 1440 Canal St, New Orleans, LA 70112 USA

**Keywords:** Corporal punishment, Latin America and the Caribbean, Intergenerational trauma, Adolescent mother, Adversity

## Abstract

**Background:**

Evidence suggests that women who experience corporal punishment as a child are more likely to use it with their children, particularly in low-resource settings where higher exposure to additional adverse childhood experiences, such as food insecurity and a parent’s premature death or abandonment, compounds damage from early exposure to corporal punishment. However, mothers who experienced corporal punishment as a child and simultaneously kind, compassionate, secure caregiving from the same or another caregiver, are less likely to expose their children to corporal punishment. Not enough research investigates how early maternal experiences with corporal punishment impact everyday parenting behaviors in contexts of poverty outside high-income countries. This investigation seeks to provide actionable information for researchers and practitioners to support families to heal from intergenerational trauma in settings of poverty.

**Methods:**

We used a convergent mixed methods design to understand how early experiences with corporal punishment shape parenting practices. We conducted a brief demographic and health questionnaire followed by in-depth semi-structured open-ended interviews with 25 mothers (19—42 years old) of low socioeconomic position in the Dominican Republic, who had children 3–5 years old at the time of the interview. Women offered reflections about how they believe their childhood experiences shaped their approach to parenting. We analyzed interview content using thematic analysis, comparing themes between women who use corporal punishment and those who do not.

**Results:**

Fourteen women reported not using corporal punishment and 11 reported using it. A large majority of all participants described receiving corporal punishment as a child (79% of those who do not use corporal punishment and 82% of those who do). Participants often struggled to remember experiences from childhood and became emotionally disconnected or desensitized when discussing abusive events from their early life.

Some participants expressed wanting to raise their children without corporal punishment, however they could not always control their impulses. A few mothers demonstrated resolution when discussing their early experiences with corporal punishment.

Adolescent mothers reported using corporal punishment with their child much more frequently than older mothers, with only one mother over the age of 20 at the birth of her first child using corporal punishment. Those who used corporal punishment with their child also reported higher rates of characteristics suggesting lower socioeconomic position. We identified two main categories for participant explanations for the use of corporal punishment: (1) a disciplinary strategy used after escalated threats, or (2) a response to feeling overwhelmed. Whether mothers considered corporal punishment a violent parenting behavior depended on whether it led to injury, what part of the body was targeted, its regularity, and whether they perceived its use was warranted.

**Conclusions:**

For early child interventions to be effective at preventing use of corporal punishment among women of low socioeconomic position in the Dominican Republic, practitioners should consider low-cost, scalable community-based therapeutic programs that address the impact of traumatic early childhood experiences.

**Supplementary Information:**

The online version contains supplementary material available at 10.1186/s12905-025-03742-y.

## Background

Women who faced and internalized adverse childhood experiences (ACEs) as a child can have heightened awareness of these events during pregnancy and postpartum, a period in life when women may experience elevated stress and preoccupation [1–3]. Early experiences, particularly for children under the age of five, play a central role in establishing a child’s network of internal neural synaptic connections that undergird the brain’s development during childhood and adolescence. These experiences create the foundation upon which the child builds skills and ultimately navigates the world as an adult. Experiences that affirm and respond to a young child’s need for safety, security, caregiving warmth and affection, and cognitive stimulation are essential to a child’s ability to thrive and achieve their full potential [4]. ACEs, such as physical and/or emotional abuse, neglect, abandonment, parent’s substance use, and/or traumatic events such as violence and hunger can negatively impact relationships, school and work achievements, mental health and behavior [5], and physical health throughout childhood, adolescence, and adulthood [6, 7].

If severe, adverse childhood experiences can be relived as trauma and manifest as fear and stress during pregnancy and later maladaptive parenting practices [8]. One systematic review, which included 97 studies that investigated associations of parental childhood maltreatment with parenting behaviors, found that parents who reported physical child abuse were more likely to engage in this behavior as parents [9]. The effect is cumulative, indicating that adults who experienced more maltreatment earlier on in their childhood are at higher risk of perpetrating child abuse [9].

Corporal punishment is one of the most common forms of violence experienced by children in the home, with about 60% of children as young as one experiencing regular corporal punishment worldwide [10]. Evidence ties the use of corporal punishment to negative early child development (ECD) and school performance outcomes [11, 12]. One longitudinal study of 2,340 families found that spanking predicted externalizing behavior with the same magnitude of association as other adverse childhood experiences such as sexual abuse, neglect, and parental substance abuse [13]. A study conducted in Chile showed that corporal punishment and negative ECD outcomes have a dose–response effect [14], a finding echoed in other studies which have also found that the impact on development intensifies with increased exposure [15].

There is currently an active debate about whether to include corporal punishment as an adverse childhood event on standardized instruments [13]. Corporal punishment behaviors vary, ranging from light spanking or slapping to regular, severe beatings [16]. Presence or absence of caregiver anger during, and child reactions to, the event also vary across social and cultural contexts. Little is known about how early maternal experiences with corporal punishment may relate to everyday parenting behaviors in contexts of poverty, outside high-income countries, where childrearing is typically understudied. A focal agenda for this study is to contribute to this debate via the use of a discourse methodology that goes beyond simple survey data, allowing for more sophisticated thematic analyses and post-hoc reflection.

In the Dominican Republic, 64% of children experience violent discipline per the 2019 Multiple Indicator Cluster Studies (MICS) survey adapted from the Parent–Child Conflict Tactics Scale [17]. The survey, conducted by the government of the Dominican Republic and UNICEF [18], asks the caregiver eleven questions about whether the child in the household has been shaken, shouted, yelled or screamed at, spanked or hit, slapped, called dumb or lazy, or beaten in the last month [19]. The survey defines violent discipline as any form of physical or psychological aggression. This prevalence of corporal punishment is similar to Luft et al. who found that 53.6% of a sample of 142 school children in the Dominican Republic reported experiencing physical abuse as children, measured as part of a standardized ACE instrument [20]. This study did not report how severe the abuse was, who was responsible for the abuse, or how the children experienced it, but did find a correlation with being a victim and perpetrator of dating violence later in life. Another study based on demographic data from 2007 in the Dominican Republic found that 28% of women on average witnessed violence from their fathers toward their mothers during childhood, and that witnessing violence predicted them experiencing intimate partner violence later in life, but it did not measure how many experienced violence as a child or used corporal punishment toward their children [21]. Regionally, a systematic review that looked at co-occurrence between intimate partner violence and corporal punishment in Latin America and the Caribbean did not include the Dominican Republic due to lack of co-occurring survey data [22].

The aim of this study is to provide an in-depth and nuanced understanding of motivation and intent around corporal punishment among mothers of low socioeconomic position in the Dominican Republic. For this paper, we define “corporal punishment” as physically punishing a child using spanking, hitting, slapping, or punching, and also locking in a room, depriving the child of food or comfort, and using threats of violence or deprivation, reflecting UNICEF’s definition [10]. Specifically, this study was framed around the following questions: first, how do mothers’ reports of their own adverse childhood experiences translate into use of corporal punishment with their child? Second, how do parents develop protective mechanisms to interrupt the cycle of adversity and shield their child from corporal punishment? Third, how does being an adolescent mother impact these experiences? Finally, we examined convergence and divergence among experiences of women who reported use of corporal punishment at the time of interview with their child and those that did not. We focused intentionally on mothers with children under the age of five because of the critical period of brain development and, thereby particular vulnerability of early childhood.

## Methods

### Design

This is a mixed methods study using a convergent design with mixing occurring at the interpretation phase [23]. We chose a convergent mixed methods design because we were interested in examining in-depth qualitative data on beliefs and practices about corporal punishment from the perspectives of mothers who inflict and do not inflict it on their children.

Quantitative and qualitative data were collected and analyzed simultaneously and considered equally in analysis. The qualitative portion of the analysis uses thematic analytical methods to identify patterns and interpret meaning within the data. Thematic analysis is useful in textual analysis that compares and contrasts personal experience and views and opinions between groups, such as in our case, between mothers who use corporal punishment with their child and those who do not [24]. The quantitative analysis uses cross-sectional methods. This approach allowed us to both explore a range of presumptions we had about the data in an open-ended fashion using inductive reasoning and triangulate data sources to increase our confidence in the results using deductive reasoning. Quantitative data was drawn from 1) a short demographic and health questionnaire and 2) counts pulled from qualitative interviews.

### Study setting

We focused on the Dominican Republic because of the high reported incidence of violence and lack of qualitative research to examine the phenomenon. Additionally, the investigators have academic collaborations in the country, including a local child development specialist, and access to a study sample.

### Recruitment and consent

This study draws from 25 interviews with adult women living in three low-income communities, with a child under five years of age. Women were from Los Mina, an urban community in East Santo Domingo, Boca Chica, a semi-urban community about 30 min east of Santo Domingo, and Yamasá, a low-income rural community about 1.5 h north of Santo Domingo populated by many people who work in Santo Domingo. Santo Domingo is the capital of the Dominican Republic and the second largest metropolitan area in the Caribbean, after Havana, Cuba, with a population of 3,245,000 [25]. Almost half (49.3%) of the children in the country live in poverty, and 7.7% in extreme poverty, according to the most recent government statistics [26].

We recruited participants from a study conducted in 2019 and funded by UNICEF called “Pregnant Women-Centered Care in the Dominican Republic Project (Project MAC)” [27]. Project MAC used childbirth observations to identify obstetric violence in two public hospitals in the Dominican Republic. All women giving birth at the time agreed to be contacted for future studies by phone. All women from the PMAC study had children born in the fall of 2019.

The PMAC contact list contained 278 contacts total, with 169 contacts living in Santo Domingo or Boca Chica, which we sorted by distance and contacted beginning with those closest to the UNIBE Neurocognition and Psychophysiology Laboratory, where the team is based. In early 2023, one of the team members called each phone number from the list and recruited women to participate in the study. If they did not answer the phone, we followed up with a text message on WhatsApp. Of the PMAC list, 112 numbers were out of service, and 36 did not answer. Of the remaining 21 women we contacted, 13 agreed to participate in interviews. Others were initially interested but did not respond to further requests to set a date for the interview [7] or reported they did not have time for the study [1]. To complement our limited sample size, we recruited an additional 12 women from Yamasá using snowballing sampling to achieve saturation on the main themes we explored. Prior to initiating the interview process, we completed an informed consent procedure with each participant, who received a compensation for their time.

### Sociodemographic questionnaire

Each woman who accepted to participate in the study completed an oral interviewer-administered questionnaire at the beginning of the data collection event, after providing informed consent. The brief questionnaire included questions about sociodemographic characteristics and health history. Sociodemographic questions included age, marital status, educational level, occupation, food security, and ages of each child or dependent. Food insecurity was evaluated with one item that asked how many times in the last four weeks the participant has worried about not having enough food in the home on a three-point Likert scale (sometimes, often, many times) [28]. The questionnaire also included yes/no questions about whether the participant had ever experienced drug or alcohol abuse, depression, chronic disease such as diabetes, and COVID- 19. An open text field at the end allowed participants to add any additional health problems requiring treatment or hospitalization. We recorded details such as treatment and hospitalizations in a free text field. Administering the questionnaire took approximately 15 min.

### Qualitative data collection

Immediately following the quantitative questionnaire and during the same encounter, members of the team facilitated a recorded semi-structured interview with open-ended questions. All participants were asked the same set of questions, and prompts were added when appropriate to enlist more detailed responses. We selected the interview questions to encourage parents to describe and reflect upon their own childhood experiences; their current parent–child relationship and interactions; and the social and economic factors that surround their family life. We organized the interview guide into three chronological sections about life experiences: 1) experiences with adult caregivers before the age of twelve, 2) experiences and circumstances of pregnancy and childbirth, and 3) current material conditions, relationship with the child’s biological father, and parenting practices (e.g., discipline, interaction behaviors with the child). We reviewed available guides and approaches to explore these phenomena and, ultimately, selected questions from the Adult Attachment Interview Protocol (AAI) and Working Model of the Child Interview (WMC).

The AAI is a semi-structured interview instrument designed by Howard and Miriam Steele to assess a person’s internal working models of attachment through narrative [29, 30]. In the AAI, a person is asked a series of open-ended questions about their experiences with childhood caregiving, attachment, and the impact of those experiences on their adult beliefs and behaviors. The narrative is then systematically coded for attachment representations, coherence, unresolved trauma or loss, and specific discourse markers by trained coders. We chose not to use the entire instrument and structured coding scheme because our goal was to identify broad themes that might inform public health practice, and we wanted to remain open to the possibility of unexpected findings. Ultimately, we incorporated 12 of 20 questions into the qualitative interview guide (numbers 1, 2, 5, 6, 8, 9, 12, 13, 14, 16, 17, and 20).

The WMC Interview can be used to understand the relationship between the child and parenting practices, particularly related to internal representations (i.e., working models) of the relationship between parent and child [31]. We used nine of ten questions from the WMC (numbers 1, 2, 3, 4, 5, 6, 7, 9, and 10). We simplified some questions structured for instrument validity (1, 5, and 6). For example, for question 6, which asks the interviewee to list five words that come to mind when thinking of their relationship with their child, we kept the original question and removed the request for five words. To further elicit self-reflection in narrative form, we asked women how they might connect their early experiences with their current parenting philosophy and practices. The qualitative interview guide is attached as Supplement 1. Participants were encouraged to use analogies and metaphors to describe their feelings and experiences.

The interview lasted an average of 60 min. The first author trained two Dominican junior psychologists as research assistants. She conducted the first five data collection events while they observed. The research assistants then facilitated data collection events with the first author present. Data collection was done in Spanish in public meeting places such as restaurants and shopping malls near participants’ homes. Because no such place was available in Yamasá, we collected data in the patio of a private home. In four cases, the PI collected data via Zoom with video because the participant had moved from Santo Domingo to a distant location. We confirmed with participants that they felt they were in a private place during the Zoom interviews. We recorded interviews, and the two research assistants transcribed them verbatim in Spanish. The bilingual (Spanish–English) PI translated quotes into English, and the bilingual research assistants reviewed each translation, editing as needed.

### Qualitative analysis

We used Dedoose to organize and assist in the analysis of the text [32]. The team co-coded five interviews separately until we achieved 80% agreement. Then two team members coded the remaining interviews, reviewing every fifth interview together to prevent drift.

We identified corporal punishment as a dominant theme during interviewing and the preliminary review of interviews. The research team then created a draft codebook based on identified themes relating to the research topic (corporal punishment), the research context, and theoretical framework (adverse childhood events and attachment theories). We refined the codebook during the open coding period until it reflected all themes [33]. We identified the following prevalent themes: 1) early experiences with corporal punishment, 2) reflections on how these experiences shaped beliefs and practices, and 3) beliefs and practices involving corporal punishment with their own child. Table 1 lists all themes and subthemes from the analysis. After we finished coding, we wrote a summary paragraph for each theme in the codebook, focusing on how participants reflected on their lived experiences using words that revealed affectation (for instance emotional response or lack thereof) or decision (for example, how an experience as a child impacted their behavior as an adult). Each summary paragraph highlighted predominant commonalities and differences in participant discourse. We then wove the themes into a narrative using thematic interpretation as described by Clarke et al. [24]. During this exercise, we took into account our team’s knowledge of Dominican childrearing culture and practices and theories of attachment. Given the sensitive nature of the topic and the interdisciplinary and international background of the analysis group, we prepared a positionality statement (Supplement 2). Finally, we considered how themes differed between mothers who do use corporal punishment and those who do not. Additional quotes by corporal punishment use are available in Supplement 3.

**Table 1:** Qualitative themes

Theme	Subtheme
Early experiences with corporal punishment	Primary caregiver bonding and rejection
	Extent of physical and emotional abuse
Reflections on how these experiences shaped current beliefs and practices	Divergent paths: embracing and departing from how they were raised
	Emotional disconnect and pathways to healing
Current beliefs and practices involving corporal punishment with their own child	How participants talk about corporal punishment
	Logic and emotions: how participants present their disciplinary approaches

### Quantitative analysis

We entered quantitative data into an excel spreadsheet exactly as it was on the questionnaire. We developed summary statistics (mean, number, and percent) using excel and reported them by participant.

### Counts derived from qualitative interviews

During qualitative and quantitative analysis, we quantified responses from the qualitative data. This included whether the participant experienced corporal punishment and whether they use it currently with their child. We then conducted a third round of analysis by separating participants into two independent groups: women who reported using corporal punishment with their child and those who did not, using Creswell’s convergent design approach [23]. We selected “yes” to corporal punishment if the participant mentioned the following terms in her interview with respect to spanking her child: *dar pela*, *darle*, or *le meto* —all of them common terms used to describe hitting someone. We also included other less commonly used phrases that came up in interviews such as *pegar* (hit, punch), *pinchar* (puncture), or *encerrar* (lock or shut someone in a space such as a room or home). We selected “no” if the participant explicitly said they did not use corporal punishment using one of those terms. We identified clear “yes” and “no” responses in each case. We also quantified questions from the qualitative data regarding experiencing corporal punishment, being neglected as a child, and having a positive or negative relationship with her mother and father, and assigned a “yes” or “no” response for each participant. For each participant, we determined her primary caregiver as a child by conducting a full review of each interview and identifying the person who spent the most time with her as a child. For instance, one question asked if they remembered with whom they spent the most time during their childhood.

### Ethics approval

Our research was conducted in accordance with the Declaration of Helsinki. We received approval for the study from the Tulane University Human Research Protection Program (# 2019–2375) and the Universidad Nacional Iberoamericana (UNIBE) Research Ethics Committee (# CEI2022 - 13).

## Results

### Participant characteristics

Table 2 presents participant characteristics stratified by use of corporal punishment with their child and organized by age at first birth. Fourteen women reported not using corporal punishment with their child and 11 reported using it. Participants were an average of 27 years old (19–39 range). Women who did not use corporal punishment were on average about 1.5 years older than the other group at the time of the interview, were more likely to have finished high school, be married or with a partner, have been raised by a mother or father as a primary caregiver, and have had a positive relationship with their mother.

**Table 2:** Characteristics of the study population by corporal punishment use

Sociodemographic characteristic	Does not use corporal punishment (*N* = 14)	Uses corporal punishment (*N* = 11)
	N (%) or mean	N (%) or mean
**Current status**		
Age at first birth	**21.2**	**17.5**
15–19	**7 (50)**	**10 (91)**
20–24	**4 (29)**	**1 (9)**
≥ 25	**3 (21)**	**0 (0)**
Number of children	**2**	**2**
1	**8 (57)**	**4 (36)**
2	**2 (14)**	**3 (28)**
3 +	**4 (29)**	**4 (36)**
Finished high school	**11 (79)**	**7 (64)**
Married or living with partner	**8 (57)**	**3 (27)**
Wage earner	**5 (36)**	**6 (55)**
Food insecure	**10 (71)**	**8 (73)**
Depression symptoms	**3 (21)**	**4 (36)**
**Childhood experiences**		
Mother/father primary caregiver	**11 (79)**	**4 (36)**
Received corporal punishment	**11 (79)**	**9 (82)**
Neglected as child	**3 (21)**	**5 (45)**
Positive relationship with mother	**7 (50)**	**4 (36)**
Positive relationship with father	**7 (50)**	**7 (64)**

Among the group who did not report using corporal punishment, there was a much lower percentage of adolescent mothers (under the age of 20) at first birth (50% versus 91%), and the mean age at first birth was higher (21.2 versus 17.5 years). Of the eleven participants in this study who reported using corporal punishment with their child, ten were adolescents when they gave birth — seven were under 18 and three were 18 or 19. The final participant who reported using corporal punishment was 21 at the time her first child was born. This contrasts with the composition of the group of 14 who did not use corporal punishment with their child—seven were adolescents–two were under 18 and five were 18 or 19—and seven were at least 20 when they first gave birth.

As seen in Table 2, a higher percentage of the group who used corporal punishment had three or more children, were living with food insecurity, earned their own wages, reported being neglected as a child and said they had a positive relationship with their father. A high percentage of both groups of women reported experiencing corporal punishment as a child (79% of non-corporal punishment users and 82% of corporal punishment users).

### Early caregiver experiences

Overall, participants were raised by their mother and father together (*N* = 7), grandparent(s) (*N* = 7), mother only (*N* = 6), aunt and uncle (*N* = 2), father only (*N* = 2), and older sister (*N* = 1) (Table 3). About half of the participants said their mothers were present in their lives as children. About one third provided positive reflections about their mothers, describing their mothers as affectionate, non-judgmental, and strong women. There was an appreciation for the selflessness and dedication demonstrated by their mothers, despite struggles or difficulties related to providing for the family. Likewise, about a third of participants had positive memories of their father, describing him as an authority figure and provider, but not affectionate or communicative.

**Table 3:** Participant characteristics by participant ID

Participant ID	Age	Age at first birth	Number of children	High school finished	Married or living with partner	Wage earner	Food insecurity	Reported symptoms of depression	Primary caregiver as child	Corporal punishment as child	Neglected as child	Positive relationship with mother	Positive relationship with father
	**Does not use corporal punishment with 3–5-year-old child (*N* = 14)**												
19	27	15	2	1	0	0	1	0	M	0	0	1	1
20	26	15	1	0	0	0	1	0	G	1	1	0	1
1	19	18	1	1	0	1	1	0	M, F	1	0	1	0
3	28	18	5	0	1	0	1	0	M	1	0	1	0
7	22	18	1	1	1	0	1	1	M, F	1	0	1	0
11	23	19	1	0	0	0	1	0	M, S	1	0	1	1
21	24	19	1	1	0	1	0	0	M, F	1	0	0	0
6	31	20	3	1	1	1	1	0	A, U	1	1	0	0
22	31	20	4	1	1	0	0	1	A, U	1	1	0	0
18	24	21	1	1	1	1	1	0	M, F	0	0	0	1
4	27	21	1	1	1	0	0	0	M, F, G	1	0	1	1
25	28	27	1	1	1	0	0	0	M, F	1	0	1	1
12	42	31	2	1	0	0	1	1	M	1	0	0	0
24	38	35	3	1	1	1	1	0	M, F	0	0	0	1
**Avg/n**	**27.8**	**21.2**	**2**	**11**	**8**	**5**	**10**	**3**	**-**	**11**	**3**	**7**	**7**
	**Uses corporal punishment with 3–5-year-old child (*N* = 11)**												
8	26	15	3	1	1	1	1	0	S	1	0	1	1
16	39	16	3	1	0	0	1	1	G	1	0	1	0
2	21	17	1	1	0	1	1	0	G	0	1	0	0
5	28	17	3	1	0	0	1	0	M	0	0	1	1
9	24	17	2	0	0	1	1	0	F	1	0	0	1
13	36	17	3	0	0	0	1	0	M, G	1	1	1	1
17	20	17	1	0	1	0	0	1	G	1	0	0	0
14	22	18	1	1	0	1	1	0	G	1	1	0	1
15	20	18	2	0	1	0	1	1	F	1	0	0	1
23	28	19	2	1	0	1	1	0	G	1	1	0	0
10	24	21	1	1	0	1	1	1	G	1	1	0	1
**Avg/n**	**26.2**	**17.5**	**2**	**7**	**3**	**6**	**8**	**4**	**-**	**9**	**5**	**4**	**7**

*M* mother, *F* father, *G* grandparent, *S* sister, *A* aunt, *U* uncle

#### Early experiences with corporal punishment

##### The extent of physical and emotional abuse

Twenty of the 25 participants reported receiving corporal punishment from caregivers when they were a child, although at least half struggled to remember any positive or negative memories before the age of 12. The most reported types of corporal punishment were physical neglect (*N* = 7) and physical punishment or abuse (*N* = 20).

*Participant #23 (adolescent at first birth who used corporal punishment with child): Yes, my father sometimes hit us. When we got into fights with others or lied, my dad would hit me. To correct my behavior. One time he beat me, he hit me so hard that my feet swelled up...*

*Participant #23: With the heel of a shoe, he hit me in my ankles.*

Participants shared reasons for receiving physical corporal punishment, including acting selfish or spoiled, losing items, not obeying parents, or lying. One participant shared that she had been beaten so much that she became numb to the experience.

*Participant #9 (adolescent at first birth who used corporal punishment with child): When I was little… my mom fought with me because I lost a key. She hit me a lot. I went to school angry… A friend bumped into me, and I grabbed her and pulled her hair, and I bit and beat her. They suspended me for a week...*

*I left her whole face… she still has scars. Later she grabbed me. She was so angry, I didn’t fight back, I let her hit me. She came and found me because she was so angry, she attacked me from behind. I was thin, but strong.*

*Interviewer: After that happened, did they scold you at home?*

*Participant #9: My dad disciplined me, and my mom beat me with a stick. She beat me a lot, I still have the scars, they haven’t left. ‘Don’t do this!’, ‘why are you so aggressive with me?’ ‘Shut up!’ and she would hit me in the mouth. And I would say, ‘hit me harder!’ and she would hit me harder ‘hit me harder!’ and she hit me even harder, until a time came that it hurt so bad. My mother beat me so much I would pass out or cry myself to sleep.*

The trauma of repeated, severe beatings led this participant to disconnect emotionally. This defense mechanism enabled her to continue on with her life in the short term, but she and her siblings lived in fear of her mother’s angry episodes. When her mother arrived home, she would routinely scold her, bite her, hit her with a belt and shoes, and throw cooking pots, ladles, and knives at her. In addition to experiences of physical punishment from her mother, some participants shared examples of emotional violence, which often came accompanied by physical abuse.

*Participant #9 (adolescent at first birth who used corporal punishment with child): Sometimes the neighbor’s kids came. We played yun [a local game], parcheesi, monopoly, cards, we went to the park, we had ice cream. But when my mother came home, everything turned black, because my mother always came home looking for a fight.*

*Interviewer: So you kept a distance?*

Participant #9: *Agrees* We were afraid of her, we are still afraid of her, but little by little it is changing.

This participant demonstrates maturity when she describes how her mother’s attacks impacted her emotionally. Other participants were not able to articulate their own emotional response as clearly.

*Interviewer: Could you tell me about a memory you have with your mother?*

*Participant #15 (adolescent at first birth who used corporal punishment with child): She talked badly about me in front of other people.*

*Interviewer: She fought with you in front others? What did she say?*

Participant #15: *Laughs* That I am a whore, she called me a bitch, a slut.

*Interviewer: How did you feel when she said those words?*

*Participant #15: Bad, my heart felt like it was breaking.*

Like physical abuse, events of emotional violence shared during interviews were mostly instigated by a female caregiver. One reported being locked in the apartment or in her room for most of her childhood (#19). Another was repeatedly accused of stealing from her stepmother. Two (#21 and #23) mentioned they were treated unfairly, compared to their sisters, being deprived of food, comfort, or companionship.

##### Primary caregiver bonding and rejection

All participants raised by their grandparents reported using corporal punishment (7/7). Among the twelve participants who reported being raised by someone other than their mother, ten reported negative early experiences with their mothers, including those quoted below. Corporal punishment use was lower among participants raised by their mother and father together (0/7). Women who were adolescents at first birth were also more often raised by grandparents (6/17) versus women who were adults at first birth (1/8).

Negative early experiences with mothers shared by participants involved the absence of their mother during their childhood *(N* = *5)*, physically and emotionally aggressive interactions *(N* = *3)*, or neglect due to always being busy *(N* = *2)*.

*Participant #9 (adolescent at first birth who used corporal punishment with child): My mom and dad fought a lot because my mom was very jealous. My mom was… they say alcoholic, right? Someone who drinks a lot. She sold everything to buy alcohol. We had no food to eat. My dad came and left her money, but finally he left the house and left us alone with my mom. We were hungry all the time. We had to work, until finally he decided to come back.*

In these cases, we saw participants heroizing another caregiver, such as the father, in their narratives. Overall, participants had higher expectations for their mothers’ involvement in their early lives compared to their fathers. In these cases, absent mothers were described as unloving, while absent fathers as busy or not communicative.

*Participant #22 (20 or older at first birth who did not use corporal punishment with child): My mom wasn’t very attentive. She had several defects that I think about now that I am a mother. I don’t want to do the things to my kids that my mom did with me…She wasn’t a very good person. She was never there, she was absent. My mother doesn’t know how to read or write, so she didn’t care about us having that privilege. She thought, ‘they can learn if they want.’*

Resentment and anger came through in cases where their mothers were absent, disengaged, or uninvolved in their lives.

#### Reflections on how these experiences shaped current beliefs and practices

##### The logic of corporal punishment use

Of the twenty participants who experienced corporal punishment as a child, nine reported using corporal punishment with their child today. Of those, two believed their own caregivers’ use was excessive, but still used it with their child, suggesting that beliefs about corporal punishment don’t necessarily guide behavior. The others believed it was warranted and helped them learn right from wrong.

Those who expressed that their parents used corporal punishment with them for their own well-being were grateful and believed their parents showed them love, due to, and sometimes despite, being strict. However, not all of them choose to do the same with their children.

*Interviewer: How was your relationship with your mother when you were little?*

*Participant #25 (20 or older at first birth who did not use corporal punishment with child): My mom was a little tougher than my dad. If my mom had to put a heavy hand on us, she would put it on us. But today I thank her, because thanks to that, I am who I am. She never stopped loving us. Even today that lady is a sweetheart, despite the fact that she laid a heavy hand on us.*

Others who expressed beliefs that corporal punishment was warranted highlighted having been hit in areas that didn’t harm them (e.g., feet or knees), only when they really deserved it, or only once or twice ever.

Of the other eleven participants who experienced corporal punishment as children but did not used it with their own children, two believed their childhood beatings were excessive and two believed they were needed, citing lessons on differentiating right from wrong. The other seven believed they were not excessive at the time but did not believe they were necessary to raise a well-disciplined child. In an example of excessive childhood beatings, participant #22 was beaten by her divorced father for saying she wanted to live with her mother. The same participant described how her mother beat her after discovering she was skipping school. Participant #22 intentionally chose not to use physical punishment with her children in contrast to what she perceived as her own cruel upbringing.

##### Divergent paths: embracing and departing from how they were raised

Overwhelmingly, women, regardless of current corporal punishment use, reported wanting to raise their child differently compared to how they were raised *(N* = *22)*. Some of these intentions were aspirational while others manifested as decisions and actions. The responses of mothers who were adolescents at first birth were more often aspirational, while mothers who were at least 20 at their first birth, particularly those that were older at the interview, reflected more resolve. When considering how they would like to change their approach to mothering, almost all (19) talked about being more loving.

*Interviewer: How do you think the experiences you told me about formed your personality as an adult?*

*Participant #23 (adolescent at first birth who used corporal punishment with child): I try not to repeat patterns, give more love to my children. I always lie down with them and talk, I tell them I love them, ‘I love you’, I practically live hugging them. I always try to be affectionate, so they feel loved. I try to make them feel the affection that I didn’t receive.*

Women considered both absent and aggressive mothers to be unloving. Other examples provided by participants about how to be more loving mothers included providing their children with a stronger family structure, more parental guidance on how to navigate difficulties in life, and greater physical presence in the home (e.g., not being as involved in nightlife). Among positive attributes inherited from their families, participants noted the value system (knowing right from wrong) and closeness with their mother.

##### Emotional disconnect and pathways to healing

Twelve mothers demonstrated dismissive discourse around their negative early experiences with caregivers. This manifested as sharing few to no childhood memories, sharing few details about the memory, sharing memories but being unable or unwilling to articulate how they were emotionally affected, or being unable or unwilling to reflect on the experiences. For example, this caregiver tried not to think about it.

*Interviewer: When you were little, did you ever feel unloved or rejected in your household?*

*Participant #19 (adolescent at the time of first birth who did not use corporal punishment with child): …. Ever since my brother was born, I always felt like that. She always… all the attention and everything was for my brother. The one who paid more attention to me was my father. But ever since my brother was born my mom would say she loved me, but I could tell that it wasn’t the same as with my brother. They loved my brother more than me.*

*Interviewer: What did you do when you felt that way?*

*Participant #19: I usually cried, I would feel sad and after a while it would go away. I don’t think about it. My dad was attentive to me.*

Dismissive discourse was particularly common among the eight participants who reported experiencing child neglect. However, five of the eight demonstrated signs of beginning to heal from their experiences. One participant (#17) was beginning to forgive her mother for being absent and consciously raising her child differently. She was able to talk about emotions from her childhood, connect them with her own childrearing beliefs, and understand how her mother’s life circumstances led to raising her in the way she did.

*Participant #17 (adolescent at the time of first birth who used corporal punishment with child): No, I don’t think it affects me, because today, since the first day that I found out I was pregnant, I said that I would give my daughter everything my mother didn’t give me. …I have always tried to show her that with me she will have more than a mother, even more than a mother because really, really, when I was little, I longed for her to at least be there, just that. I always told my daughter that I will always be there for her… she is very young, but I always tell her. She is very intelligent, she always looks for a way to brighten up my days. She is very open, she knows a lot.*

*Interviewer: Well, thinking about this with your mom, now that you're an adult. Have you thought about why she did what she did? Have you thought about it from her perspective?*

*Participant #17: Yes, sometimes I think about it and then I say to myself that, well, she loves me in her own way and I have to love her like that. She's my mother, she was the one who brought me into the world and I have to love her anyway and I have to respect her and love her as she is. But I try not to give it that much thought, and I try to cling to the fact that I already have my own daughter, and that I have to fight for her and try to give her everything. That ever since she came into my life, the best thing that God could have sent me in my life was her, because of her I don't feel alone, and I always cling to her… she always gives me a reason to keep going and keep fighting for her.*

Another participant (#22) who experienced and witnessed severe physical abuse as a child described the experience and how she was impacted. In this excerpt, she showed her ability to transmit information about her parents’ behavior, including the injustice she felt about their anger with her, and her perception of their behavior as self-consumed. Her laughing may have demonstrated some dismissiveness, perhaps in the context of sharing emotional information with someone with whom she had not developed extensive trust.

*Interviewer: So, in the first house did you feel loved, or did you feel rejected?*

*Participant #22 (20 or older at first birth who did not use corporal punishment with child): Not in the first house, I mean, my dad lived near there. I witnessed many fights, mommy and daddy were always fighting because daddy wanted me to live with him and mommy wanted me to live with her. They were always in a fight, never showing affection. They never took into account, first, what does our girl feel, what does she need? No, they were fighting among themselves:"I want her to live with me!"and so on.*

*Interviewer: And you were just there watching.*

*Participant #22: Yeah, it was like a ball. Back and forth, back and forth, and so on.*

#### Beliefs and practices involving corporal punishment with their own children at the time of interview

##### How participants talked about corporal punishment

Participants used general terms to describe the behaviors we classify here as corporal punishment, such as *le meto* or *le doy una pela*, which translate generally to spanking, hitting, slapping, or beating the child with something, such as a sandal or belt. Most participants did not detail their behaviors even when probed, however, two mothers specifically described pushing their child. One mother talked about *hincar*: putting her child on her knees and holding her hands above her head for an extended time.

##### Logic and emotions: how participants presented their disciplinary approaches

When asked what child behaviors prompted a physical response from caregivers as mothers, participants reported spoiled, stubborn, impatient, or aggressive behavior, breaking something of value, not obeying, having a tantrum in public, or crying too much.

Participants fell into two categories: those who used logic to defend their choice to use corporal punishment as a disciplinary strategy (n = 7), and those who reported reacting to a strong emotional impulse they couldn’t control (n = 6). Two participants fell into both categories.

The seven participants with a logical discourse emphasized that they used corporal punishment only when they believed that the child deserved it. They reported an escalated disciplinary approach beginning with a verbal request (e.g., change a specific behavior), then a threat of harm if they did not comply, and finally the use of corporal punishment if the child’s behavior persisted. These mothers commonly talked about the use of corporal punishment as a last resort or because they had no choice due to the severity of the child’s behavior.

*Participant #2 (adolescent at the time of first birth who used corporal punishment with child): I gave her a beating in the last few days, because she smashed and hit the television screen. And she made a mess. And I hit her. When she breaks stuff and things like that, I hit her. And when she hits me, too, sometimes she makes me mad. Sometimes she wants to poke me with something sharp, and I tell her:''If you poke me, I'll hit you”. And, if she pokes me, I hit her. But she's not doing that stuff as much as before [...] But I don't hit her just because. I don’t just hit her for any little thing, only when I see she deserves it. When I see that I can't handle her anymore. When she breaks something or hits me. If I speak to her with authority and she doesn't listen to me, I hit her. Because if I don't do that, she'll go out of control. With the age she is, if I just left her like this, imagine what it will be like when she's five years old...*

In contrast, six participants used corporal punishment because they felt overwhelmed by the child’s behavior and were unable to control their emotional response when the child acted out. All participants who discussed using physical punishment with their child out of anger or impulse also expressed regretting it later.

Interviewer: How do you think your behavior affects the child? Participant #8 (adolescent at the time of first birth who used corporal punishment with child): At first, I felt like it affected him negatively because I honestly couldn’t control myself, I didn’t know how to manage situations. Sometimes I even pushed him. One time, I pushed him off the bed. But thank God, I learned to manage it with time. I learned to love him more. Because I am a woman who doesn’t like to fight *laughing*. That is what happened with him. Honestly, I never have fought much. I raised my nephews, but since they were not mine, if they started fighting, I would just pass them off to their mother. But since he is mine, I have to put up with it, because he is mine. With time I have tried to project something positive. Earlier, he didn’t want anything to do with me, he was attached to his father. I think that’s why, but now I see him, and he is attached to us both. However, when he is with his father, he also looks for me. Before, no. Before, he only went looking for his father. Honestly, his father hit him too, but not how I did. I used to hit him cruelly, with bad intentions.

Mothers like this responded emotionally out of fear or anger. They may have been unconsciously modeling behavior from their own caregivers, trying to exert power over their child in response to feeling out of control, or projecting an image of control to other adults, such as the child’s father. Their regret suggests a sensitivity to the child’s feelings and experiences and an awareness of other non-violent and effective disciplinary strategies, but a difficulty aligning their behaviors with their beliefs. Some participants were unable or unwilling to modify their behavior, others, like participant #8, had been able to change their behavior over time with enormous effort.

Finally, this participant distinguished between hitting with cruelty and hitting for disciplinary purposes only, suggesting the former was more acceptable. This distinction appeared in discourse a few times and, although the sample is small, seems to align with the distinction of an emotional, or angry response versus a calculated, logical disciplinary strategy.

Of the fourteen caregivers who reported not using corporal punishment, eight said they actively chose a non-violent childrearing approach. Two mentioned observing other mothers who promoted corporal punishment, again demonstrating an awareness of different opinions about the subject.

*Participant #18 (20 or older at first birth who did not use corporal punishment with child): Sometimes I wish someone would tell me what to do, like an intelligent person. There are people who tell me:"You grab her and give her beating and she will behave well”. I don't want to mistreat her because, if in my house I was raised without mistreatment, raised well, I shouldn’t mistreat her.*

This participant expressed doubt in her ability to control her child but demonstrated the contrary in her decision not to follow commonly held beliefs. Another three participants mentioned they believed corporal punishment was ineffective and cruel and that there were other ways to promote discipline.

*Participant #6 (20 or older at first birth who did not use corporal punishment with child): I don't judge them. In fact, there are mothers who, when their children are naughty, they think it's because [the child] is asking for it. I don't question their judgement. It’s a process. But it's not because [the child] was asking for it. Peeing in the bathroom, if it came out accidentally, he was asleep and he thought he was in the bathroom, I just change the sheets and put him in a corner. But, there are mothers who hit him, and I think that is wrong. Especially when the child is fast asleep. What does the child know? He is not going to say"Mommy, why are you hitting me? I was sleeping, I didn’t know better". Because when a person is asleep, they were probably half asleep, half awake. He doesn’t know better.*

Some mothers shared alternatives to corporal punishment, which included speaking in an authoritative tone, having the child stand on their knees for a long period of time, telling them the police were coming, threatening to, but not actually hitting the child, and taking away toys or other privileges. Some perceived that having the child stand on their knees for a long period of time was not a form of corporal punishment.

## Discussion

We sought to understand the use of corporal punishment among low-income mothers in the Dominican Republic and how beliefs and practices at the time of interview may have been shaped by their own early experiences receiving corporal punishment.

Findings indicate the normalization of violence in the study population, with perspectives of corporal punishment as a valid approach to discipline and an emphasis on a nuanced, continuum vision, wherein corporal punishment ranged from very light punishment to severe abuse. Several qualifying factors expressed by participants, including severity (whether it causes physical injury), whether or not it was warranted (how bad the behavior was that prompted its use), and the regularity of use determined whether they considered a behavior corporal punishment. Participants suggested that bodily strikes that didn’t hurt, didn’t leave a mark, or were only threats do not constitute corporal punishment. These beliefs were reflected in their own experience as children as well. Some mothers responded initially that they did not receive any corporal punishment, but later conditioned their statement by saying that it was either deserved, did not happen regularly, or did not hurt. In almost all narratives, the sense of ‘hurt’ was only ascribed to the physical, disregarding ways that corporal punishment can operate emotionally to create rage, a sense of betrayal, a tacit sense of permission to use hitting as a way of interacting, feelings of rejection, and feelings of disrespect towards the parent.

The 2019 MICS Survey findings echo our findings showing a high prevalence and acceptance of corporal punishment for discipline: 9% of caregivers believed that physical punishment was necessary to discipline a child, and among those with lower education and socioeconomic position, this number increased to 12–15%, a figure more reflective of the demographics of this study sample [17]. Research that defines corporal punishment across cultural contexts is underdeveloped. However, views similar to those expressed by mothers from this study have been documented worldwide, including among studies conducted in Latin America and the Caribbean [34, 35]. One study administered a questionnaire to adults in Barbados and found that 70% approved of corporal punishment and agreed that it was unwarranted only when parents used punishment in an unsystematic, excessive, or self-serving manner [35].

Studies have shown that beliefs about corporal punishment do not always dictate behavior among parents who experienced physical abuse or trauma as a child [9]. One qualitative study among adolescent mothers in New Orleans similarly found a misalignment between discourse and behavior with respect to other behaviors, such as contraception use [36]. In our case, about half of the women who wished not to use corporal punishment with their children reported doing so. This may be due to impulsive behavior stemming from unresolved trauma and could be a topic for future research [37]. When interpreting prevalence statistics, researchers and practitioners should be aware that beliefs and expectations about corporal punishment do not necessarily align with behavioral practices.

Despite about half of the cohort being raised by a family member other than her mother, the most severe reports of emotional and physical neglect and physical abuse experienced by participants when they were children were those perpetrated by their mothers. This finding corroborates findings by Luft et al. which indicate that perpetrators of physical child abuse are more likely to be mothers compared to fathers [20]. There are a couple of possible explanations for this. Our research suggests higher parenting expectations from mothers versus fathers. Normative beliefs in settings such as the Dominican Republic with high gender inequity position mothers as the loving, affectionate, and self-sacrificial caregiver and fathers as the caregivers expected to “keep order.” Because in this setting use of corporal punishment is a stark deviation from the behaviors expected and desired of a mother, its incidence may produce a stronger negative reaction and memory than it would coming from the father. Alternatively, it may reflect mothers spending more time with children than fathers. In the Dominican Republic, about 35% of children are raised in mother-only households [38]. With limited support and resources for childrearing, mothers may experience high levels of stress that increase the likelihood of resorting to corporal punishment to keep order. While fathers on the contrary, may spend only a few hours with the child and with that time develop a positive, stimulating relationship or memories.

A few participants who appeared to be working through adverse childhood experiences based on their ability to talk about them with some objectivity, shared emotions related to the events, told the stories in chronological order, related a theory of how they influenced them as children, and articulated their own different approach to motherhood, potentially demonstrating the first steps toward finding resolution of past trauma. Caregivers’ wishes about promoting their children’s future well-being and their expressed restraint and remorse about resorting to corporal punishment in their interactions with their children provided sources of hope and strength in their future ability to cope with daily stressors of living in poverty.

Women in our study were carrying a double burden of motherhood, in some cases beginning in adolescences, and poverty. Similar to our findings of high rates of corporal punishment use among adolescent mothers, findings from the national 2019 MICS survey administered by UNICEF and the Dominican government, indicated that younger mothers were more likely to believe that corporal punishment was necessary to raise a child compared to older mothers [17]. This may be due, first, to adolescent mothers being poorer than older mothers. Our quantitative results suggest that mothers who first gave birth as adolescents were experiencing more poverty than those who first gave birth at 20 or later, indicated by their higher rate of food insecurity, lower education levels, higher report of neglect, and fewer ties to their mothers and fathers as their primary caregivers as a child. Both adolescent motherhood and poverty have been shown to lead to an increase in corporal punishment use in studies in the region. In a large cross-sectional study in Juiz de Fora, Brazil, younger age and lower socioeconomic position increased the odds of corporal punishment use among women [39]. While we did not measure adverse childhood experiences (ACEs) using a standardized instrument, it is likely that many in this group experienced multiple ACEs as a child, leading to emotional pain and trauma.

Second, feeling underprepared to be a mother adds to the stress of experiencing intense hormonal and behavioral changes characteristic of adolescence, such as a need for peer approval, experimentation with intimate relationships and sexuality, and identity definition through self-discovery. Research shows that, compared to mothers who have their first child at a later age, adolescent mothers have less parenting experience, less education, less financial means, are under increased stress, and have higher risk of partner violence [22]. Another qualitative study conducted with the same sample of 25 mothers demonstrated how the multiple stressors of adolescent motherhood lead mothers to feel they lack the maturity, tools, and experiences to discipline their children [40]. In the Dominican Republic, where 15.3% of children were born to mothers aged 15 to 19 in 2021 [41], grandparents often care for the child, the higher rate of grandparents as primary caregivers among the group that corporally punished their child is a signal for concern.

Third, attachment theory provides insight into how a child’s distress can ignite a mother’s past conflicting attachment and defense system, resulting in unintended “fight, flight, or freeze” behavior [37, 42]. A mother with unresolved trauma may expose her child to neglectful, violent, or inconsistent caregiving through maladaptive responses to events perceived as dangerous or unimportant [43]. A parent’s hypervigilance and/or avoidance can interfere with healthy interactions between a caregiver and a child. As a result, maladaptive interaction patterns may look like a lack of responsiveness to the child, or may manifest as a parent’s intrusive efforts to overly control, admonish, and constrict a child’s behavior [44]. This could explain cases where participants report acting impulsively when the child cries, whines, or hits such as reports cited above from participants #2 and #8.

We found evidence of avoidant narratives throughout the interviews. When participants were asked to reflect on past events, many said they just chose not to think about it. This could be due to either blocked memory due to trauma, as described by Crittenden in their analysis of insecure attachment-avoidant style [45], an avoidant coping mechanism, or hesitancy to share this information with the interviewer.

While it was not possible to clinically assess trauma in this study, some experiences reported during the interviews suggest severe neglect and physical abuse were present in this cohort. This shows that research studies can identify prevalence of clinically relevant trauma and that the use of corporal punishment with children may provide behavioral indicators of such. Interventional research can inform the design of therapeutic approaches by psychologists and trauma-trained case managers to address the long-term stress and mental health of future generations [43, 46, 47].

### Limitations

Many participants were unable to talk about early child experiences before the age of 12, so many of the experiences described here occurred in later childhood. This may be because the topic was too sensitive to gain trust with interviewees over the course of only one one-hour interview, or that fear that reporting their own physical punishment before 12 could stigmatize or penalize their caretaker. Therefore, it is possible that our study underreports the prevalence of corporal punishment perpetration and victimization due to reporting bias. Due to the small sample size, we could not detect statistical associations between quantitative variables and in turn could not determine whether adolescent mothers are more likely to use corporal punishment with their children. Future quantitative studies could investigate statistical associations.

## Conclusion

In this study, we set out to describe how mothers believed their early experiences with corporal punishment impacted their beliefs and practices in caring for their children. Findings suggest that a mother’s age at first birth and cultural norms may drive participants’ beliefs and practices. Many mothers expressed wanting to be more loving and less aggressive than their caregivers were with them, but also indicated they were not always able to do so with their children. Further research in the Dominican Republic must take into account culturally appropriate definitions of events when measuring prevalence using standardized instruments. Women from the Dominican Republic may not report corporal punishment if they are asked simply whether they use physical punishment with their children.

We found the use of corporal punishment was greater among adolescent mothers compared to older mothers in our sample. Within broader social targets, prevention programs should focus their attention on adolescent women. First, women need resources to prevent pregnancy when they do not want to become pregnant. Second, they need resources to continue their studies or to develop marketable skills. Third, should they become pregnant, they need support to enhance their parenting skills, to learn about the impact of early experience on child development, and to develop social support systems for parenting. Parenting is often taught as an individual skill, but programs should formally recognize the role of older kin and other family members in childrearing, especially for female-headed households. Such approaches can take advantage of cash transfer programs and other support mechanisms and provide young women with opportunities to develop careers.

Published research in the field of psychotherapy shows that mothers who are in the process of understanding the impact of early trauma on their current assumptions and behaviors change their parenting practices first, even before fully resolving the trauma [43]. Ultimately, our goal is to help public health practitioners and researchers identify when and how maternal adverse childhood experiences may present a risk to children [43] and further inform the design of effective intervention approaches to reduce corporal punishment and increase positive and responsive parenting behaviors that promote healthy child development. Prevention programs focused on the resolution of early trauma by helping mothers become aware of it and training them to address it could be a promising approach to preventing violence and interrupting the intergenerational transmission of trauma. Low-cost, community-based, scalable therapeutic efforts, such as the Minding the Baby home-visiting program [48], have proved to prevent child mistreatment and could be adapted or developed for this setting. Significant work needs to be done to create these programs specifically for families in the Dominican Republic.

## Supplementary Information

Supplementary Material 1

Supplementary Material 2

Supplementary Material 3

## Data Availability

The datasets generated and/or analyzed during the current study are not publicly available due the data being qualitative and not fully de-identified, but are available from the corresponding author upon reasonable request.

## Abbreviations

ECD: Early child development
ACEs: Adverse childhood experiences
