# Supplementary material for: Adverse childhood experiences and use of corporal punishment among women in low-resource settings: a convergent mixed methods study with mothers of children under five in the Dominican Republic
Source: BMC Womens Health. 2025 Dec 3;25:591. doi: 10.1186/s12905-025-03742-y (PMC12676820; doi:10.1186/s12905-025-03742-y)
Supplement: Supplementary file 1 — Supplementary Material 1 [file 12905_2025_3742_MOESM1_ESM.docx]

**Supplement 1: In-depth interview guide**

**Principal Investigator:** Ms. Katrina Nelson

**Thesis Advisor:** Dr. Arachu Castro

**Co-investigator:** Dr. Laura Sánchez-Vincitore

Participant code: _____________ Interview date: day __ month __ year __

Interviewer: __________________________ Location: ____________________________

**Family history in childhood and adolescence**

1. Where were you born and on what date?

2. During your childhood and adolescence, who lived in your home?

3. What did the people in your household work for/how did they earn a living?

4. Did your parents go to school? Until what grade level?

5. Did you work when you were little?

6. How many brothers and sisters did you grow up with and how many are older or younger than you?

7. In this household, how many people slept in the same room?

8. How did the people in your home get along?

9. Did they talk to you about sexuality at home? What about contraception?

10. Did you go to school? / Do you go to school?

11. What education level did achieve? / What level are you in now?

12. If you have dropped out of school, why?

**Sexuality and pregnancy**

1. How did you find out that you got pregnant?

2. How old were you / your partner?

3. How many weeks were you when you found out you were pregnant?

4. What was your reaction when you found out?

5. Who did you tell about the pregnancy first?

6. Did you want to get pregnant?

7. Had you used a condom or some other type of contraceptive method to prevent your first pregnancy?

8. Have you had any miscarriages? Tell me about them.

9. Who supported you during your pregnancy?

10. When did you receive medical care?

11. Did you share any concerns you had about the pregnancy with the doctor?

12. Were you given/prescribed vitamins? Have you been able to take them? If not, why?

**Relationship**

1. How did your relationship with your child's father change as a result of the pregnancy?

2. Are you still in contact with him?

3. Did you have to face new difficulties as a result of the pregnancy? Which?

**Current home**

1. Who lives in your current household?

2. What do they do/how do they earn a living?

3. In this household, how many people sleep in the same room?

4. Do the children in this household go to school?

5. How do the people in your household get along?

6. What do you like most about your home?

7. What do you like least about your home?

**Your child(ren)**

1. How many children do you have? How old are they?

*Now we are going to focus on (child's name, who was born in 2018).*

1. What is (*child's name*) like?

2. Explain to me what an ordinary day in the life of (*child's name*) would be like.

3. Does he/her go to school? How many people take care of him/her?

4. What are the dreams you have for your child?

5. Do you have any concerns about your child's nutrition?

6. Do you have any concerns about your child's health and development?

**Mother-child interaction**

1. What are the activities you most enjoy doing with your child?

2. What are the values ​​you want to pass on to your child?

3. What are the concerns you have about your relationship with your child?

4. Are you satisfied with how you interact with your child, or is there something you would like to change?

5. And with respect to the other caregivers in your life, are you satisfied with how they interact with your child? What do you like or dislike?

**Own life**

1. Regarding your life, have you achieved what you set out to do?

2. What are your future plans and how have they changed?

3. Are there any other comments you would like to make?
