# Supplementary material for: Adverse childhood experiences and use of corporal punishment among women in low-resource settings: a convergent mixed methods study with mothers of children under five in the Dominican Republic
Source: BMC Womens Health. 2025 Dec 3;25:591. doi: 10.1186/s12905-025-03742-y (PMC12676820; doi:10.1186/s12905-025-03742-y)
Supplement: Supplementary file 2 — Supplementary Material 2 [file 12905_2025_3742_MOESM2_ESM.docx]

**Supplement 2. Positionality statement**

The Nurturing Care Framework (NCF), a ‘roadmap for action’ launched by the World Health Organization, UNICEF, and the World Bank in 2018 (at the 71st World Health Assembly), is based on ‘state-of-the-art evidence about how early childhood development can be supported and improved across the globe.’ In addition to articulating young children’s needs for healthy nutrition, protection from harm and disease, and opportunities to explore, play, and learn, the NCF asserts that all young children need ‘responsive caregiving’, characterized by caregiver-child interactions that ‘respond to the child’s signals and needs’, and that promote ‘safe and mutually-rewarding relationships between caregiver(s) and child’ (1). Exploring childrearing behavior and establishing benchmarks to track children’s capacity to thrive requires an in-depth understanding of how caregiving occurs and varies in different socio-economic, cultural, and geographical contexts. These types of analyses are necessary in order to 1) prevent misunderstandings of the nature of caregiving, and 2) prevent underestimating the development of children from low-income and/or less well-studied settings. Both issues can lead to the creation of well-intentioned yet misguided interventions (2, 3).

This qualitative exploration of corporal punishment and its use as a threat, a widespread childrearing practice in the Dominican Republic, is intended to help provide a roadmap to design culturally affirmative interventions that protect young children from harm. A. Katrina Nelson designed and conducted this study as part of her requirements to obtain her doctoral degree in public health from the Celia Scott Weatherhead School of Public Health and Tropical Medicine at Tulane University.

Nelson, who is also fluent in Spanish and has extensive experience conducting research in Latin America, conducted the study in the Dominican Republic, building on the existing research collaboration on early childhood development between Arachu Castro (professor at Tulane University and doctoral committee chair) and Laura Sánchez-Vincitore (professor at Universidad Iberoamericana in Santo Domingo and doctoral committee member). While in the Dominican Republic, Nelson trained Dominican psychology students Michelle Susana and Melanie Frías in qualitative data analysis. Sánchez-Vincitore, Susana, and Frías provided data interpretation and contextual feedback on the content of this study. Nelson was also advised by her other doctoral committee members at Tulane: professors Katherine Theall and Carl Kendall. Additionally, Nelson invited Martha Vibbert (clinical associate professor in psychiatry and pediatrics at Boston University School of Medicine) and Heidi Luft (assistant professor at University of Texas Medical Branch), given their international research experience and expertise relevant to the topic.

**References**

1. WHO. Nurturing care for early childhood development: a framework for helping children survive and thrive to transform health and human potential. Geneva: World Health Organization, United Nations Children's Fund, World Bank Group; 2018.

2. Scheidecker G, Chaudhary N, Keller H, Mezzenzana F, Lancy DF. "Poor brain development" in the global South? Challenging the science of early childhood interventions. Ethos. 2023.

3. Scheidecker G, Chaudhary N, Oppong S, Rottger-Rossler B, Keller H. Different is not deficient: respecting diversity in early childhood development. Lancet Child Adolesc Health. 2022;6(12):e24-e5.
