# Supplementary material for: Adverse childhood experiences and use of corporal punishment among women in low-resource settings: a convergent mixed methods study with mothers of children under five in the Dominican Republic
Source: BMC Womens Health. 2025 Dec 3;25:591. doi: 10.1186/s12905-025-03742-y (PMC12676820; doi:10.1186/s12905-025-03742-y)
Supplement: Supplementary file 3 — Supplementary Material 3 [file 12905_2025_3742_MOESM3_ESM.docx]

| ***Theme*** | ***Uses corporal punishment*** | ***Does not use corporal punishment*** |
| --- | --- | --- |
| ***Use of corporal punishment with child*** | | |
| *Beliefs* | *Interviewer: What do you do when he doesn’t want to go to school?*  *Participant #10: I punish him, I tell him I'm going to take away his tablet and things like that. *Laughs**  *Interviewer: Does what you do work to correct his behavior?*  *Participant #10: Yes, yes, it works for me because he cannot be without the tablet. When I tell him that I am going to take it away from him, he calms down.*  *Interviewer: Ok, did you ever take it away?*  *Participant #10: Yes, now with the storms going on, I have to take it to get checked because it turned off and won’t turn back on. I took it to the technician and he told me to bring it back tomorrow. Tomorrow I'm going to take it back to see what’s going on.*  *Interviewer: Ok, so now it's not working. Have you ever had to take it away because [name of son] misbehaved?*  *Participant #10: All the time.*  *Interviewer: And does he remember?*  *Participant #10: Yes.*  *Interviewer: What does he do when you take it away? Does he cry, or throw a tantrum?*  *Participant #10: Yes, he starts to cry.*  *Interviewer: What do you do when he cries?*  *Participant #10: I hit him sometimes, but not much, because daddy will say, "don't hit the boy!"* | *Participant #6: When he says something inappropriate, I say, “Papi, who told you that? You don’t do that. I don’t say that. You don’t hear me saying that.” I talk to him like an adult, and I speak clearly to him so he understands and can learn. “You don’t do this, boom boom.” I don’t do this. Because he isn’t a child who wants problems. If he is upset, it is usually because someone bothered him. When he is aggressive with another child, it is because that child did something to him. For instance, he wanted to take a toy away from him. You know, they are like animals. For example, animals don’t use language, and what happens? Animals attack first because it’s their defense, and children are the same way. I don’t judge, in fact, there are mothers who, when their child misbehaves, think it is because they want to. I don’t judge them, but it is a process, it is not because they want to. Or when they go pee in the bathroom, and they went pee on the floor by accident, they were half asleep, and they thought it was the bathroom. I just change the sheets, and maybe have him sit in a corner, but some mothers hit the child for that, and I don’t think that is correct, especially if the child is half asleep. How will they know? The child will not say, “Mommy, why did you hit me? I was so sleepy, and I didn’t know what I was doing”.* |
| *Practices* | *Participant #8: The crybaby, that makes me very angry *Laughs*. I can't hear a child crying much. I try to control myself. A while ago I couldn’t control myself, but with the passage of time I have learned how to control myself and I cope better. When he was a baby I really couldn't deal with it. I used to push him, I pushed him. I know that was bad, it was very bad, because he cried about everything. Since it was my first time, I didn't know how to handle it. I'm not very close to children, you know? With him I have learned to feel affection for children, but not all children. Not like some women who love children, it has to be my family or a child that catches my attention.* | *Participant #7: Well, at home I show him love, I show him a lot of affection. He shows me too, he shows me a lot of affection. Although I have to correct him, I have to show a stern face, but after I correct him I give him love. From the moment he gets up: "Good morning, my love, how do you feel?" he hugs me. Everything is love, that child everything is a love. Love, affection and a lot of attention for him.* |
| ***Early experiences with corporal punishment*** | | |
| *Who raised them* | *Interviewer: And your grandparents? Let's talk a little about each one. Your grandfather, first, what was your relationship with your grandfather like? Was it different than your relationship with your uncle?*  *Participant #17: Look, it was different because you know old people from before are a little different, strict. Because he was a person who was very affectionate, very kind and everything, but you had to go along with it, because you know that he would give you a hard time about anything. When you throw a tantrum, for example. But in reality it was love and he loved me so much. He was the best. He always said that he had, he has 17 children and he said that he has 18 children. Apart from those who have already died, he said that he had 18 children and that I was his youngest daughter. Although I was very naughty; he would hit me because I was very naughty when I was little. But he played with me and was a sweetheart with my daughter too. Every time I went to visit him and didn't take my daughter, he would get mad, because I didn't take the girl. Just love.* | *Interviewer: How would you describe your relationship with him when you were little? With your uncle.*  *Participant #6: Well, my uncle has always been, and still today is, a peasant. He hurt me a lot, but I understand now that perhaps it was his way of correcting me. Maybe now someone would see him and say, "he mistreated her, we have to take him to a psychologist, call the police." But that was the way of educating children back then.*  *Interviewer: All the children got that.*  *Participant #6: You know? Now, things have been modernized and updated. If they see that you hit a child, they are going to call the police, the psychologist, the child. It was a way to parent. At that time, a child could not go out, not even to the corner.* |
| *Experiences* | *Participant #9: He hit me once, but I think it was because I asked him for money in front of people. He didn't like it, he told me: "Come, I’m going to give you a beating", inside the kitchen he hit me a lot and then left and I cried. That was the only time my dad gave it to me.* | *Interviewer: When you were little, did you ever misbehave?*  *Participant #20: Oh, I used to go and hide from my mom sometimes in the river area *Laughs*. My mom didn't like that, she gave me hell for it. *Laughs* I would go and hide, even knowing she would hit me I would hide *Laughs*. Yes, she didn't like any of that.*  *Interviewer: And sometimes when she hit you, was it too much or was it ok?*  *Participant #20: No, it always seemed normal to me. Sometimes she just got over it. I would leave her alone and go away until she got over it. I would hide and then sneak into bed *Laughs* and she wouldn’t do anything to us, she got over it.*  *Interviewer: *Laughs**  *Participant #20: Yes, but there were times when she would grab me, she would be waiting for me behind the door and when I entered, she would hit me with the belt *Laughs*.*  *Interviewer: How old were you when that?*  *Participant #20: About 9, 11 years old, more or less. She didn't like it because of that, because on the other side of the river was Gualey. All along the river was dangerous. She never liked me going. There were always men, they were crazy “tigueros” (street guys), she didn't like it. I went to that area with friends, she never liked it. I would hide from her, it wasn't always, just a few times, but she didn't like it.* |
| *Beliefs* | *Interviewer: At some point, when you misbehaved, did your mother and father threaten you with a beating, punishments?*  *Participant #21: Yes, they gave me two or three beatings, but they weren't that strong. They always took away the TV, that was tough. But nothing very severe.*  *Interviewer: Would you say that when they punished you or hit or beat you, was it excessive?*  *Participant #21: No, they were things like hitting us with a flip-flop. I would be crying before they even hit me. Then they would just tell me there is no TV for you. I would have to came home from school, go to class in the morning and that's it, no games, nothing. It was never anything very severe.* | *Interviewer: Did they ever physically hurt you? A beating...*  *Participant #14: No, they wouldn’t do it without a reason, like there are some parents who give their children beatings for the sake of it. If I did something that deserved a beating, yes, or a punishment, something like that, yes, they gave it to me.*  *Interviewer: Did it happen frequently?*  *Participant #14: No, not often.*  *Interviewer: Only if you misbehaved.*  *Participant #14: Very badly, yes.*  *Interviewer: Do you remember any experiences where you behaved very badly?*  *Participant #14: That I behaved very badly and they gave me a beating... No, I don't remember, because that was when I was very little, because they never did when I was in adolescence.* |
| ***Impact of early experiences on current beliefs and practices*** | | |
| *Processing early experiences as an adult* | *Participant #17: He gave me shit or punished me like making me kneel on the wall.*  *Interviewer: How did that work? Was he correcting your behavior that way?*  *Participant #17: Huh?*  *Interviewer: Did the punishment work?*  *Participant #17: Of course, yes, thanks to him I am what I am and today. I am not a person who does wrong things or anything like that.*  *Interviewer: Do you ever think he did something that was unfair or shouldn't have been done?*  *Participant #17: No, because of him and because of his way of being I am who I am. Because of how he raised me, I am now a person who does not abuse my child, but I try to teach her the good things and to lead her on the right path. Because of him, I never did the wrong thing or anything like that.* | *Participant #18: No, I never experienced someone giving me a beating as a child, but I always had respect [for my parents] because, who wants to go for an hour with their hands up? *Laughs**  *Interviewer: Terrible for a child. Participant #18: But I didn't see that as a terrible punishment, because I had friends nearby and I saw that the punishments that their parents gave them were ugly and strong. In my mind I said this was nothing *Laughs*. An example, I really liked television, we all liked it, what he did was he said that no one was going to watch television. I felt that was a harsh punishment, like they were taking away what I liked. There in the house we had balls, hoops and he said that no one was going to play with those toys. My mother, mainly, said that today we are not playing ball, we are not going to dance hoops, we just have to sit there. I did feel that it was a strong punishment because they took away what I liked.*  *Interviewer: And boredom *Laughs**  *Participant #18: And apart from boredom, he made us read. It made me want to cry, I felt like that was a terrible punishment.* |
| *Parenting intentions* | *Participant #9: [The beatings] affected me because I don't want to be like that, but I am sometimes. I can be aggressive, and I can take things the wrong way. I try to control myself; I try not to be [like my mother] because I don't want to be like her, but sometimes I take things the wrong way, and I get aggressive, I want to fight, I want to scream, but I control myself. Sometimes I can't control myself. Sometimes I can be very aggressive, sometimes a bit aggressive with the children too, that's why I sent them to live with my parents. The same things my mother did to me, I was starting to do with them. Biting their hands so they would be quiet; when I wanted them to be quiet, I would bite their hands. I thought, “Please God, forgive me.” It wasn't what I wanted; I don't know why I did it.* | *Interviewer: Is there anything you learned in your childhood that you are applying with your children?*  *Participant #16: I don't like being hit, hitting scares me. The blows irritate me, I don't like to lay my hands on anyone because the blows irritate me, you know. There are people who like to lay their hands on children, I don't like to hit or punch. That’s why I tell [my children], I tell them: "Don't let anyone lay their hands on you, don’t let anyone hit you, but you don't do it either."* |
